# Supplementary material for: Tissue-specific (ts)CRISPR as an efficient strategy for in vivo screening in Drosophila
Source: Nat Commun. 2019 May 8;10:2113. doi: 10.1038/s41467-019-10140-0 (PMC6506539; doi:10.1038/s41467-019-10140-0)
Supplement: Supplementary file 1 — Supplementary Information [file 41467_2019_10140_MOESM1_ESM.pdf]

Supplementary Information

Tissue-specific (ts)CRISPR as an efficient strategy for *in vivo* screening in *Drosophila*

Meltzer et al.

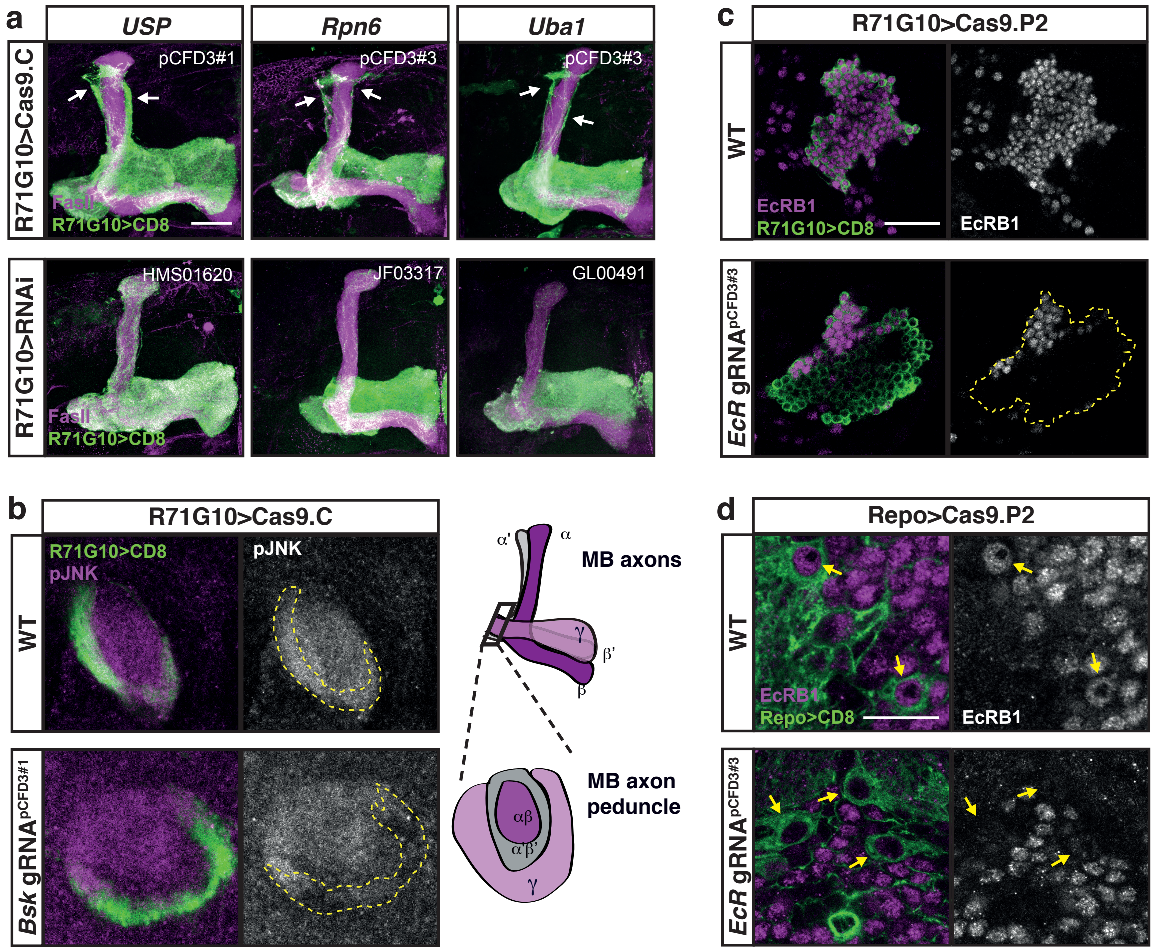

Supplementary Figure 1. Additional characterization of tsCRISPR

a. Additional examples of confocal Z-projections of adult MBs expressing the indicated gRNAs as well as UAS-Cas9.C and CD8 driven by R71G10-GAL4 (upper row); or MBs expressing the indicated TRiP RNAi's and CD8 driven by R71G10-GAL4 (lower row). White arrows highlight unpruned γ axons CD8, green; FasII, magenta.

b. (Left) Single confocal sections of the axon peduncle region of WT or Bsk-gRNA-expressing adult MBs, stained for phospho-JNK (pJNK), with UAS-Cas9.C and CD8 driven by R71G10-GAL4. CD8, green; pJNK, magenta or grey. (Right) schematic representation of the MB peduncle. Neurons grow in a concentric manner such that the first-born γ neurons are located in the periphery (adapted from ref. 4).

c. Single confocal sections of the cell body region of WT or EcR-gRNA-expressing MBs at 0h APF, stained for EcRB1, with UAS-Cas9.P2 and CD8 driven by R71G10-GAL4. CD8, green; EcRB1, magenta or grey.

d. Single confocal sections of a proximal region in the ventral nerve cord of WT or EcR-gRNA-expressing glial cell bodies at 0h APF, stained for EcRB1, with UAS-Cas9.P2 and CD8 driven by Repo-GAL4. Yellow arrows highlight glial cell bodies. CD8, green; EcRB1, magenta or grey. Scale bar represents 15 μm.

Unless stated otherwise, the scale bar represents 30 μm. Yellow dashed lines demarcate the Cas9 expression domain.

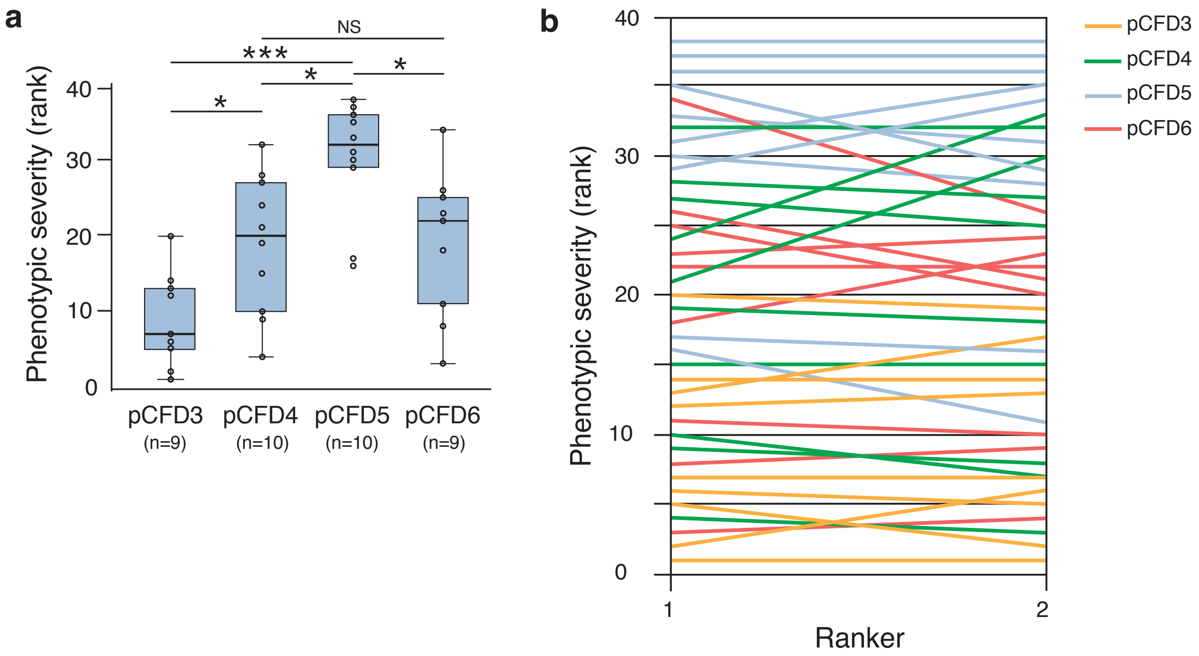

Supplementary Figure 2. Second quantification of plasmid efficiency

a. Relating to Fig. 2a,c: Boxplot depicting the second ranking of severity of the plum phenotype using pCFD3/4/5/6. Kruskal-Wallis test: $\chi^2_{(3)}=17.53$, $p<0.001$; Pairwise Wilcoxon test (FDR correction): pCFD3-pCFD4: *p=0.03; pCFD4-pCFD5: *p=0.02; pCFD3-pCFD5: ***p<0.001; pCFD5-pCFD6: *p=0.033. The box represents 1st to 3rd quartiles, whiskers represent minimum and maximum values that are within 1.5 x interquartile range, horizontal line represents the median, and empty circles represent all values within the group

b. Spaghetti-plot comparing the severity ranking by the two independent rankers. Paired Wilcoxon signed-rank test: W=249, p=0.499.

Source data are provided as a Source Data file.

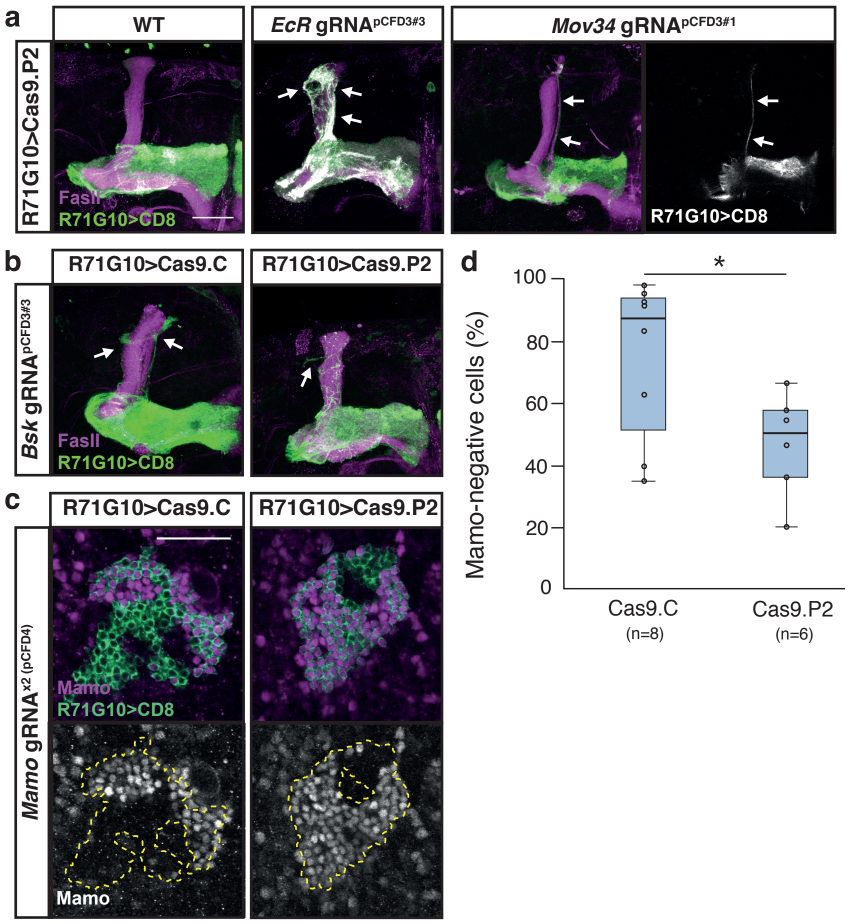

Supplementary Fig. 3. Cas9.P2 is less potent than Cas9.C and may reduce lethality

a. Confocal Z-projections of adult MBs expressing the indicated gRNAs as well as UAS-Cas9.P2 and CD8 driven by R71G10-GAL4. These specific gRNA lines were lethal using UAS-Cas9.C. For Mov34, the CD8 channel is also shown separately to highlight unpruned γ axons. CD8, green or grey; FasII, magenta.

b. Confocal Z-projections of adult Bsk-gRNA-expressing MBs with R71G10-GAL4 driving CD8 and either UAS-Cas9.C or UAS-Cas9.P2. CD8, green; FasII, magenta.

c. Single confocal sections of the cell body region of 6h APF MBs, expressing Mamo-gRNAs (in pCFD4), with R71G10-GAL4 driving CD8 and either UAS-Cas9.C or UAS-Cas9.P2. CD8, green; Mamo, magenta or grey.

d. Quantification of (c): Boxplot depicting the proportion of γ cell bodies that lost immunoreactivity to Mamo in Mamo-tsCRISPR MBs using either Cas9.C or Cas9.P2. The box represents 1st to 3rd quartiles, whiskers represent minimum and maximum values within 1.5 x interquartile range, horizontal line represents the median, and empty circles represent all values in the group. Student's t test: $T_{(12)}$=2.31, *p=0.04.

In all confocal images, scale bar represents 30μm. White arrows highlight unpruned γ axons. Yellow dashed lines demarcate the Cas9 expression domain.

Source data are provided as a Source Data file.

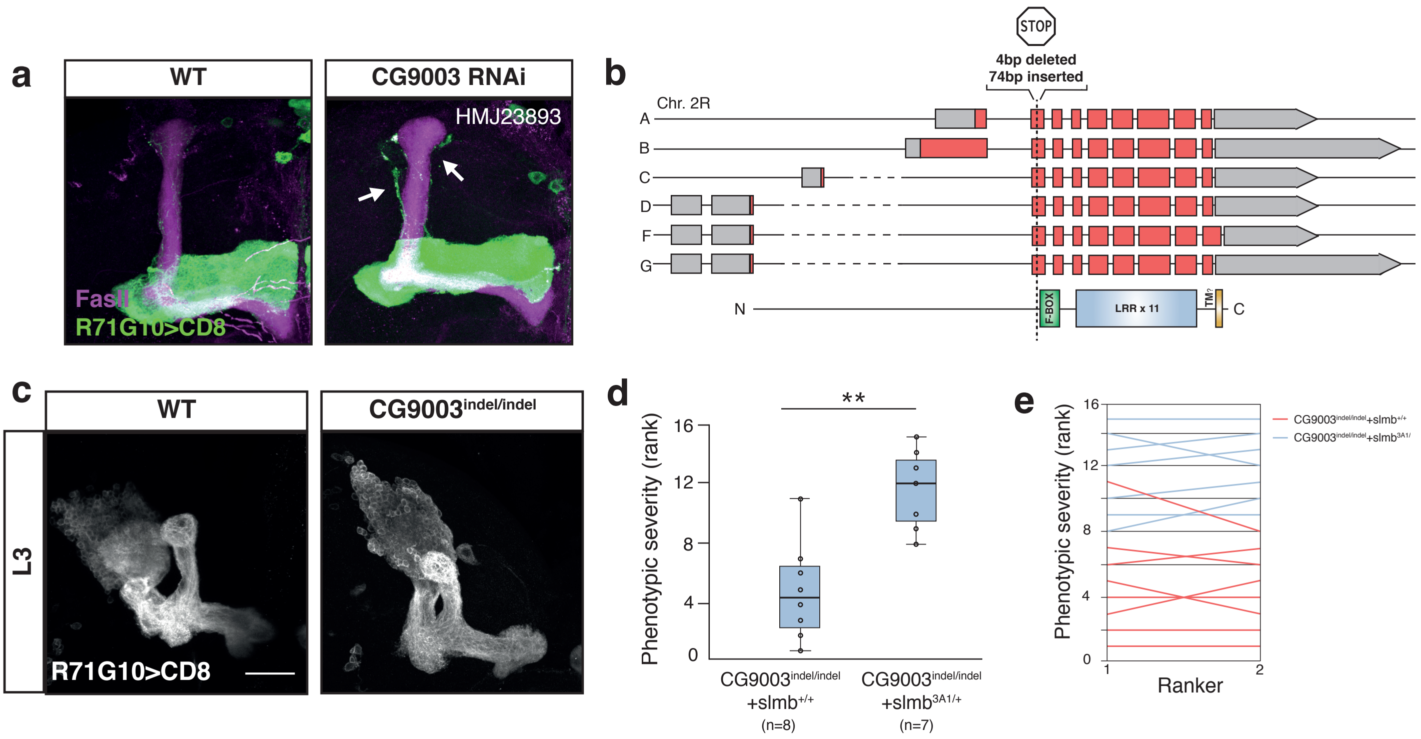

Supplementary Figure 4. Further characterization of the CG9003 phenotype

a. Confocal Z-projections of adult MBs that are either WT or express RNAi (TRiP.HMJ23893) targeting CG9003, as well as CD8 driven by R71G10-GAL4. Unpruned γ axons are highlighted by white arrows. CD8, green; FasII, magenta.

b. Schematic representation of the $CG9003^{indel}$ allele. The Different CG9003 isoforms are displayed, with red and grey blocks representing coding and non-coding exons, respectively, and black lines representing introns. The double strand break occurred at the beginning of the 2nd coding exon (15 nucleotides downstream of the translation initiation site of the coding sequence of isoform C), and led to deletion of 4 nucleotides and insertion of 74 others. The first 3 nucleotides of the 74 encode a stop codon (TAA), resulting in predicted truncation of the protein upstream of all putative domains, including the F-BOX and all LRRs.

c. Confocal Z-projections of 3rd instar larvae (L3) MBs which are either WT or homozygous for $CG9003^{indel}$. CD8 is driven by R71G10-GAL4 (grey).

d. Boxplot depicting the second ranking of pruning defect severity of $CG9003^{indel}$ homozygotes without or with $slmb^{3A1}$ heterozygosity. The box represents the 1st to 3rd quartiles, whiskers represent minimum and maximum values within 1.5 x interquartile range, the horizontal line represents the median, and empty circles represent all values within the group. Mann-Whitney U test: W=3, **p=0.002.

e. Spaghetti-plot comparing the severity ranking by the two independent rankers. Paired Wilcoxon signed-rank test: W=28, p=1.

In all confocal images, scale bar represents 30 μm.

Source data are provided as a Source Data file.

Gene symbol	Ref.	gRNA line		RNAi line	
		Target sequence	Phenotypic penetrance	#BDSC	Phenotypic penetrance
EcR	[1]	(1) TCCTCTTGCACCCGTGGCGC<br>(2) AGGCCCTGTCGCCGCAAACC<br>(3) GCAAGAAGGGACCTGCGCCA	Lethal<br>Lethal<br>Lethal	*TRiP.HMJ22371* (#58286)<br>*TRiP.HMC03114* (#50712)	100% (n=24/24)<br>11% (n=2/18)
Usp	[1]	(1) TCAGCCGAAAGCTGGCGTCC<br>(2) CTTTCGGCTGAGCCACATCA<br>(3) GCTGAGCCACATCAAGGAGG	100% (n=4/4)<br>6% (n=1/16)<br>67% (n=12/18)	*TRiP.JF02546* (#27258)<br>*TRiP.HMS01620* (#36729)	9% (n=2/22)<br>0% (n=0/17)
Plum	[2]	(1) CAATCAATTGAATCACAAAG<br>(2) GAGTCGACAACAGGTGAGTG<br>(3) GTTCTTCGGTTGGGCGACGG	61% (n=11/18)<br>68% (n=15/22)<br>64% (n=9/14)	*TRiP.HMC05055* (#60062)	43% (n=6/14)
UVRAG	[3]	(1) GTTCCGCAGTCGAAGTTGCT<br>(2) TGAATCCCTGAATTCTGTTA<br>(3) GTACGGACGACGAGGCCTGG	100% (n=20/20)<br>14% (n=3/22)<br>92% (n=11/12)	*TRiP.HMS01357* (#34368)	0% (n=0/23)
Bsk	[4]	(1) AACACTACACCGTCGAGGTG<br>(2) GTTGGTGTCCCCCACCTCGA<br>(3) GCACCAACACTACACCGTCG	75% (n=9/12)<br>83% (n=10/12)<br>71% (n=24/34)	*TRiP.HMS04479* (#57035)<br>*TRiP.HMS00777* (#32977)	35% (n=7/20)<br>0% (n=0/14)
Eip75B	[5,6]	(1) CAACAGCAGCAATCGAGCCT<br>(2) GAAGAAACTCAAATGCATGG<br>(3) GCGGCTGCAACATCATCCGG	52% (n=11/21)<br>14% (n=2/14)<br>50% (n=11/22)	*TRiP.GLC01418* (#43231)<br>*TRiP.HMS01530* (#35780)	5% (n=1/22)<br>17% (n=3/18)
Uba	[7]	(1) CGCACGATTCTTGCCGATAT<br>(2) CGATATCGGCAAGAATCGTG<br>(3) ACTGACCAACTCCGACGGGG	50% (n=12/24)<br>60% (n=6/10)<br>73% (n=19/26)	*TRiP.JF01977* (#25957)<br>*TRiP.GL00491* (#36307)	0% (n=0/23)<br>0% (n=0/7)
Rpn6	[7]	(1) GATTCACGCTGGACAATGCC<br>(2) GGAGGGCGCCGAGAACGATG<br>(3) GGGCGAGCTCTACAAGCAGG	7% (n=2/28)<br>50% (n=6/12)<br>73% (n=9/26)	*TRiP.JF03317* (#29385)	0% (n=0/14)
Mov34/ Rpn8	[7]	(1) TTTACGCTCACCTCCTGCGA<br>(2) CAAAGTGATAGTGCATCCAT<br>(3) GAAGGGCACTGGAAGTCGGG	Lethal<br>Lethal<br>Lethal	*TRiP.JF01140* (#31567)<br>*TRiP.GL00333* (#35411)	39% (n=7/18)<br>78% (n=14/18)

Supplementary Table 1. Phenotypic penetrance of gRNA and RNAi lines in proof-of-concept study

Oligonucleotide name	Forward	Reverse
Sequencing of *CG9003* indel	CTCAGAAACCCACTCGAATCG	AGCGATGTCGCGGTTTTCTCA
gRNA cloning into pCFD3[8]<br>(e.g., *Mamo*)	GTCG-20nt gRNA sequence<br>(GTCGAGTACGAGGAACAAGCCGAG)	AAAC-20nt gRNA sequence rev comp<br>(AAACCTCGGCTTGTTCCTCGTACTCGAC)
Cloning gRNAs into pCFD4[8]<br>(e.g., *Mamo*)	TATATAGGAAAGATATCCGGGTGAACTTCG-20nt 1st<br>gRNA sequence-GTTTTAGAGCTAGAAATAGCAAG<br>(TATATAGGAAAGATATCCGGGTGAACTTCGAGTACGA<br>GGAACAAGCCGAGGTTTTAGAGCTAGAAATAGCAAG)	ATTTTAACTTGCTATTTCTAGCTCTAAAAC-20nt 2nd gRNA<br>sequence rev comp-CGACGTTAAATTGAAAATAGGTC<br>(ATTTTAACTTGCTATTTCTAGCTCTAAAACCAAGCAGTAGT<br>GCTCACTGCCGACGTTAAATTGAAAATAGGTC)
Cloning *Mamo*-gRNAs into pCFD5[9]	GCGGCCCGGGTTCGATTCCCGGCCGATGCAAGTACG<br>AGGAACAAGCCGAGGTTTTAGAGCTAGAAATAGCAAG	ATTTTAACTTGCTATTTCTAGCTCTAAAACCAAGCAGTAGT<br>GCTCACTGTGCACCAGCCGGGAATCGAACCC
Cloning *Plum*-gRNAs into pCFD5	GCGGCCCGGGTTCGATTCCCGGCCGATGCACAATCA<br>ATTGAATCACAAAGGTTTTAGAGCTAGAAATAGCAAG	ATTTTAACTTGCTATTTCTAGCTCTAAAACCCGTCGCCCAA<br>CCGAAGAACTGCACCAGCCGGGAATCGAACCC
Cloning *Mamo*-gRNAs into pCFD6[9]	CGGCCCGGGTTCGATTCCCGGCCGATGCAAGTACGA<br>GGAACAAGCCGAGGTTTCAGAGCTATGCTGGAAAC	ATTTTAACTTGCTATTTCTAGCTCTAAAACCAAGCAGTAGT<br>GCTCACTGTGCACCAGCCGGGAATCGAACCC
Cloning *Plum*-gRNAs in pCFD6	CGGCCCGGGTTCGATTCCCGGCCGATGCACAATCAA<br>TTGAATCACAAAGGTTTCAGAGCTATGCTGGAAAC	ATTTTAACTTGCTATTTCTAGCTCTAAAACCCGTCGCCCAA<br>CCGAAGAACTGCACCAGCCGGGAATCGAACCC

Supplementary Table 2. List of oligonucleotides used in this study

Oligonucleotides used in this study are listed. For primers used for cloning into pCFD3 and pCFD4, the template of the primer is given and *Mamo* is used as an example. 'Rev comp' indicates reverse complement. Within the primer, the 20-nucleotide gRNA sequence in underlined. Please refer to Supplementary Table 1 and Supplementary Data 1 for a list of all gRNA sequences cloned into pCFD3 and pCFD4, respectively.

Primer design for cloning of additional gRNA sequences into pCFD5 (excluding *Mamo* and *Plum*, see list in Supplementary Data 1) was done using the proprietary services of BioBasic.

Supplementary references

1 Lee, T., Marticke, S., Sung, C., Robinow, S. & Luo, L. Cell-autonomous requirement of the USP/EcR-B ecdysone receptor for mushroom body neuronal remodeling in Drosophila. *Neuron* **28**, 807-818 (2000).

2 Yu, X. M. *et al.* Plum, an immunoglobulin superfamily protein, regulates axon pruning by facilitating TGF-beta signaling. *Neuron* **78**, 456-468, doi:10.1016/j.neuron.2013.03.004 (2013).

3 Issman-Zecharya, N. & Schuldiner, O. The PI3K class III complex promotes axon pruning by downregulating a Ptc-derived signal via endosome-lysosomal degradation. *Dev Cell* **31**, 461-473, doi:10.1016/j.devcel.2014.10.013 (2014).

4 Bornstein, B. *et al.* Developmental Axon Pruning Requires Destabilization of Cell Adhesion by JNK Signaling. *Neuron* **88**, 926-940, doi:10.1016/j.neuron.2015.10.023 (2015).

5 Alyagor, I. *et al.* Combining Developmental and Perturbation-Seq Uncovers Transcriptional Modules Orchestrating Neuronal Remodeling. *Dev Cell* **47**, 38-52 e36, doi:10.1016/j.devcel.2018.09.013 (2018).

6 Rabinovich, D., Yaniv, S. P., Alyagor, I. & Schuldiner, O. Nitric oxide as a switching mechanism between axon degeneration and regeneration during developmental remodeling. *Cell* (2016).

7 Watts, R. J., Hoopfer, E. D. & Luo, L. Axon pruning during Drosophila metamorphosis: evidence for local degeneration and requirement of the ubiquitin-proteasome system. *Neuron* **38**, 871-885 (2003).

8 Port, F., Chen, H. M., Lee, T. & Bullock, S. L. Optimized CRISPR/Cas tools for efficient germline and somatic genome engineering in Drosophila. *Proc Natl Acad Sci U S A* **111**, E2967-2976, doi:10.1073/pnas.1405500111 (2014).

9 Port, F. & Bullock, S. L. Augmenting CRISPR applications in Drosophila with tRNA-flanked sgRNAs. *Nat Methods* **13**, 852-854, doi:10.1038/nmeth.3972 (2016).
