# Supplementary material for: Tissue-specific (ts)CRISPR as an efficient strategy for in vivo screening in Drosophila
Source: Nat Commun. 2019 May 8;10:2113. doi: 10.1038/s41467-019-10140-0 (PMC6506539; doi:10.1038/s41467-019-10140-0)
Supplement: Supplementary file 3 — Reporting Summary [file 41467_2019_10140_MOESM3_ESM.pdf]

nature research

Corresponding author(s): Oren Schuldiner

Last updated by author(s): 2019/01/19

Reporting Summary

Nature Research wishes to improve the reproducibility of the work that we publish. This form provides structure for consistency and transparency in reporting. For further information on Nature Research policies, see Authors & Referees and the Editorial Policy Checklist.

Statistics

For all statistical analyses, confirm that the following items are present in the figure legend, table legend, main text, or Methods section.

n/a	Confirmed	
☐	☒	The exact sample size (*n*) for each experimental group/condition, given as a discrete number and unit of measurement
☐	☒	A statement on whether measurements were taken from distinct samples or whether the same sample was measured repeatedly
☐	☒	The statistical test(s) used AND whether they are one- or two-sided<br>*Only common tests should be described solely by name; describe more complex techniques in the Methods section.*
☒	☐	A description of all covariates tested
☐	☒	A description of any assumptions or corrections, such as tests of normality and adjustment for multiple comparisons
☒	☐	A full description of the statistical parameters including central tendency (e.g. means) or other basic estimates (e.g. regression coefficient) AND variation (e.g. standard deviation) or associated estimates of uncertainty (e.g. confidence intervals)
☒	☐	For null hypothesis testing, the test statistic (e.g. *F*, *t*, *r*) with confidence intervals, effect sizes, degrees of freedom and *P* value noted<br>*Give P values as exact values whenever suitable.*
☒	☐	For Bayesian analysis, information on the choice of priors and Markov chain Monte Carlo settings
☒	☐	For hierarchical and complex designs, identification of the appropriate level for tests and full reporting of outcomes
☒	☐	Estimates of effect sizes (e.g. Cohen's *d*, Pearson's *r*), indicating how they were calculated

Our web collection on statistics for biologists contains articles on many of the points above.

Software and code

Policy information about availability of computer code

Data collection	No software was used for data collection
Data analysis	Image analysis was performed using ImageJ v1.51n (NIH)

For manuscripts utilizing custom algorithms or software that are central to the research but not yet described in published literature, software must be made available to editors/reviewers. We strongly encourage code deposition in a community repository (e.g. GitHub). See the Nature Research guidelines for submitting code & software for further information.

Data

Policy information about availability of data

All manuscripts must include a data availability statement. This statement should provide the following information, where applicable:

- Accession codes, unique identifiers, or web links for publicly available datasets
- A list of figures that have associated raw data
- A description of any restrictions on data availability

The data that support the findings of this study are available from the authors on reasonable request

Field-specific reporting

Please select the one below that is the best fit for your research. If you are not sure, read the appropriate sections before making your selection.

☒ Life sciences ☐ Behavioural & social sciences ☐ Ecological, evolutionary & environmental sciences

For a reference copy of the document with all sections, see nature.com/documents/nr-reporting-summary-flat.pdf

Life sciences study design

All studies must disclose on these points even when the disclosure is negative.

Sample size	The differences are qualitative. We strove to image an n of ~10 independent representative brains in each group.
Data exclusions	No data were excluded
Replication	Each fly is a replicate in essence due to the independent genetic pertubation. Deviations and quantifications are reported.
Randomization	Based on classical genetic markers
Blinding	In ranking quantification, samples were blindly ranked by an independent investigator

Reporting for specific materials, systems and methods

We require information from authors about some types of materials, experimental systems and methods used in many studies. Here, indicate whether each material, system or method listed is relevant to your study. If you are not sure if a list item applies to your research, read the appropriate section before selecting a response.

Materials & experimental systems

n/a	Involved in the study	
☐	☒	Antibodies
☒	☐	Eukaryotic cell lines
☒	☐	Palaeontology
☐	☒	Animals and other organisms
☒	☐	Human research participants
☒	☐	Clinical data

Methods

n/a	Involved in the study	
☒	☐	ChIP-seq
☒	☐	Flow cytometry
☒	☐	MRI-based neuroimaging

Antibodies

Antibodies used	Primary antibodies: chicken anti-GFP 1:500 (GFP-1020; AVES), mouse anti-FasII 1:25 (1D4; DSHB), mouse anti-EcRB1 1:25 (AD4.4; DSHB), rabbit anti-active-JNK (pJNK) 1:200 (V7931; Promega) and rabbit anti-Mamo 1:5000 (Alyagor et al. 2018 Dev Cell). Secondary antibodies: FITC donkey anti-chicken 1:300 (703-095-155; Jackson immunoresearch), Alexa fluor 647 goat anti-mouse 1:300 (A-21236; Invitrogen) and Alexa fluor 647 goat anti-rabbit 1:300 (A-21236; Invitrogen)
Validation	All the above antibodies are commercially available and routinely used by our lab and many others. The Rabbit anti-Mamo antibody was generated by our lab and thoroughly validated (see Alyagor et al. 2018 Dev Cell).

Animals and other organisms

Policy information about studies involving animals; ARRIVE guidelines recommended for reporting animal research

Laboratory animals	Drosophila melanogaster, males and females
Wild animals	This study did not involve wild animals
Field-collected samples	This study did not involve samples collected from the field
Ethics oversight	This study was approved by the Weizmann Institute of Science Recombinant DNA Committee

Note that full information on the approval of the study protocol must also be provided in the manuscript.
