# Supplementary material for: Tissue-specific (ts)CRISPR as an efficient strategy for in vivo screening in Drosophila
Source: Nat Commun. 2019 May 8;10:2113. doi: 10.1038/s41467-019-10140-0 (PMC6506539; doi:10.1038/s41467-019-10140-0)
Supplement: Supplementary file 4 — Description of Additional Supplementary Files [file 41467_2019_10140_MOESM4_ESM.docx]

**Title:** Supplementary data file 1
**Description:** gRNA plasmid library and transgenic fly collection Sequences used for gRNA design, the plasmid backbone and genomic integration location (if a transgenic fly exists) are depicted.
